# Supplementary material for: Complexome profiling of the Chlamydomonas psb28 mutant reveals TEF5 as an early PSII assembly factor
Source: Plant Cell. 2025 Mar 17;37(6):koaf055. doi: 10.1093/plcell/koaf055 (PMC12164590; doi:10.1093/plcell/koaf055)
Supplement: koaf055_Supplementary_Data [file koaf055_supplementary_data.zip › psb28_interactive.zip!/plots/intensity/qualityPlots/peptides/ATP15_Cre10.g420700.t1.2.html]

### Cre10.g420700.t1.2

Trivial name: ATP15  
SeqCoverage [median]: 61.30  
Mass: 8.27 kDa  
  
 

| Sequence | MissedCleavage | Mass [Da] | wtIntensity | muIntensity | Score |
| --- | --- | --- | --- | --- | --- |
| AVMKEPFK | 1 | 948.51 | 5324.82 | 9629.55 | 153.78 |
| AVMKEPFKTK | 2 | 1177.65 | 412.10 | 3349.46 | 94.82 |
| QGVATLLETTGMPK | 0 | 1444.76 | 18666.15 | 33520.88 | 216.84 |
| QTVYFR | 0 | 812.42 | 10393.28 | 1947.77 | 92.32 |
| STPVSDGK | 0 | 789.39 | 11025.52 | 18031.65 | 87.18 |
| STPVSDGKQGVATLLETTGMPK | 1 | 2216.14 | 9842.65 | 26247.53 | 132.60 |
| VAGMSYVR | 0 | 881.44 | 14472.49 | 6696.39 | 108.09 |
|  |  |  | 70137.0 | 99423.2 |  |
